# *ETV6* Molecular Heterogeneity in Salivary Secretory Carcinoma: A Case Series Report and Literature Review

**DOI:** 10.1007/s12105-024-01673-y

**Published:** 2024-08-05

**Authors:** Farzana Mahomed, Jana de Bruin, Sizakele Ngwenya, Zinhle Masango, Katherine Hodkinson

**Affiliations:** 1 Department of Oral Pathology, School of Oral Health Sciences, Faculty of Health Sciences, University of the Witwatersrand, Private Bag 3, Johannesburg, 2050 South Africa; 2 Department of Anatomical Pathology, School of Pathology, Faculty of Health Sciences, University of the Witwatersrand, Johannesburg, South Africa; 3 Department of Molecular Medicine and Haematology, National Health Laboratory Services, School of Pathology, Faculty of Health Sciences, University of the Witwatersrand, Johannesburg, South Africa

**Keywords:** Secretory carcinoma, Salivary gland, *ETV6*, Atypical FISH pattern

## Abstract

**Background:**

*ETV6* gene rearrangement is the molecular hallmark of secretory carcinoma (SC), however; the nature, frequency, and clinical implications of atypical *ETV6* signal patterns by fluorescence in situ hybridization (FISH) has not yet been systematically evaluated in salivary gland neoplasms.

**Methods:**

The clinical, histopathologic, immunohistochemical and molecular features of seven salivary SCs, including four cases with atypical *ETV6* FISH patterns, were retrospectively analyzed along with a critical appraisal of the literature on unbalanced *ETV6* break-apart in SCs.

**Results:**

The patients were four males and three females (31–70 years-old). Five presented with a painless neck mass and two patients with recurrent disease had a history of a previously diagnosed acinic cell carcinoma of the buccal mucosa. Histologically, there were varied combinations of microcystic, papillary, tubular, and solid patterns. All tumors were diffusely positive for S100 and/or SOX10, while 2 cases also showed luminal DOG1 staining. Rearrangement of the *ETV6* locus was confirmed in 5/7 cases, of which 3 cases showed classic break-apart signals, 1 case further demonstrated duplication of the *ETV6* 5`end and the other loss of one copy of *ETV6*. Two cases harbored *ETV6* deletion without rearrangement. Two of the 4 cases with atypical *ETV6* FISH patterns represented recurrent tumors, one with widespread skeletal muscle involvement, bone and lymphovascular invasion. Surgical treatment resulted in gross-total resection in all 7 cases, with a median follow up of 9.5 months post-surgery for primary (*n* = 3) and recurrent disease (*n* = 1).

**Conclusion:**

Duplication of the distal/telomeric *ETV6* probe represented the most common (26/40; 65%) variant *ETV6* break-apart FISH pattern in salivary SC reported in the literature and appears indicative of an aggressive clinical course.

## Introduction

Secretory carcinoma (SC) of the salivary glands, previously classified as mammary analogue secretory carcinoma, is a rare and distinctive neoplasm, which typically harbors a recurrent balanced chromosomal translocation t(12;15) (p13;q25) *ETV6::NTRK3* [1]. Morphologically, salivary SCs closely resemble zymogen granule poor and intercalated duct-rich acinic cell carcinomas [2], but usually express various markers of breast SC including mammaglobin, S100 protein, SOX10 and GCDFP-15 [3]. Molecular testing is the gold standard for confirmation of the diagnosis, particularly if the morphology and immunohistochemistry findings are equivocal [4]. An *ETV6* rearrangement may be detected on paraffin embedded tissue by fluorescence in situ hybridization (FISH) with a break-apart probe and has been the laboratory method most widely used in salivary SC [5]. Alternatively, the fusion transcript can be identified by reverse transcriptase polymerase chain reaction [5], with the caveat that the *ETV6* gene may fuse with genes other than *NTRK3* [6], or that the *ETV6::NTRK3* fusion gene may display atypical exon junctions [4], or rarely harbor alternative non-*ETV6* chimeric fusions as identified by next generation sequencing [7]. Further, atypical FISH signal patterns have also been described with the *ETV6* and *NTRK3* break-apart probes in salivary SCs [8, 9]. The authors present a series of salivary gland malignancies with assessment for rearrangement of the *ETV6* (12p13) locus using a break-apart probe and review the literature on salivary gland tumors with an atypical *ETV6* FISH result.

## Materials and Methods

Seven patients diagnosed with salivary SCs between 2017 and 2023, with either a typical or an atypical pattern on FISH for *ETV6* rearrangement, were identified from the archives of the Departments of Oral and Anatomical Pathology, University of the Witwatersrand and National Health Laboratory Service, Johannesburg, South Africa. Patient clinical information, histopathologic reports and immunohistochemical expression profile for S100 protein and/or SOX10 and DOG1 were reviewed. FISH analysis was performed as part of the routine diagnostic work-up using the Vysis LSI Dual Colour *ETV6* break-apart rearrangement probe. A normal nucleus shows two fusion signals while a case positive for rearrangement of *ETV6* shows one fusion, one orange and one green signal. The orange probe spans the 5’telomeric end of the *ETV6* gene from intron 2 and the green probe flanks the 3’centromeric region of the *ETV6* gene. A minimum of 100 interphase nuclei were screened for each of the cases and images were captured using BioView Duet imaging system (BioView Ltd., Israel and Abbott Molecular, Des Plaines, IL, US). The cut-off value for positive *ETV6* rearrangement was a minimum of 10% of all analyzed cells. Institutional ethics approval was obtained from the University of the Witwatersrand Human Research Ethics Committee (M221094).

### Literature Review

We searched PubMed, Scopus and Web of Science to find relevant English-language articles (including reviews, meta-analyses, original papers, case reports, and case series) published between 2010 and 2023. Search terms included “salivary secretory carcinoma” and “mammary analogue secretory carcinoma”. The following information was extracted from each selected study (when available): the study’s publication year and country, the number of reported cases, patients’ demographic details, tumor location, final diagnosis, and details regarding the cytogenetic findings following FISH analysis with an *ETV6* break-apart probe.

## Results

### Case Series

#### Clinical Characteristics

The age of the cohort ranged from 31 to 70 years (median of 36 years), with 4 males and 3 females. The tumor involved the parotid gland in 4 cases, the submandibular gland in 1 case, and the buccal mucosa in 2 cases, with the tumor size ranging from 2.3 to 6 cm (median of 3.0 cm). Most patients presented with a painless neck mass of variable duration (ranging from 2 months to 4 years). Two of the patients had a history of a previously diagnosed acinic cell carcinoma, in the same region of the current tumor that had been previously treated by surgical excision at 9- and 10-years respectively (Table 1).

**Table 1:** Clinico-pathologic and molecular features of the currently reported cases

Case #	Age (yr) / Sex	Location	Size (cm)	S100	DOG1	SOX10	*ETV6* FISH pattern	Adverse /other histopathologic findings	Treatment	Follow-up
1	47 F	Parotid	2.3	+	-	+	60% typical (1F1O1G) pattern	None	Excision, neck LND	LFU
2	70 F	Buccal mucosa	2.1	+	+	+	100% loss of one copy of *ETV6*	None. Original diagnosis: AciCC	Excision	Recurrence 9-years post initial excision, LFU
3	36 M	Parotid	3.0	+	-	+	30% typical pattern, 10% loss of one copy of *ETV6*	None	Excision	NED (9 months)
4	32 M	Parotid	4.0	ND	+	+	75% typical pattern	None	Excision	LFU
5	35 M	Parotid	2.5	+	-	ND	85% typical pattern	PNI, nodal metastasis	Excision, RT	NED (10 months)
6	31 F	Submandibular gland	4.8	+	-	ND	20% loss of one copy of *ETV6*	None	Excision	NED (6 year, 11 months)
7	40 M	Buccal mucosa	6.0	+	-	+	60% typical pattern, of which 75% showed extra orange signals	PNI, LVI, skeletal muscle and bone invasion, nodal metastasis. Original diagnosis: AciCC	Excision, neck LND, RT	Recurrence 10-years post initial excision, NED (8 months)

FISH, fluorescence in situ hybridization; 1F1O1G, one fusion, one orange and one green signal; F, female; M, male; LND, lymph node dissection; LFU, lost to follow-up; NED, no evidence of disease; ND, not done; RT, radiotherapy; PNI, perineural invasion; LVI, lymphovascular invasion; AciCC, acinic cell carcinoma; +, positive; -, negative

### Morphologic, Immunophenotypic, and Molecular Characteristics

**Fig. 1:**
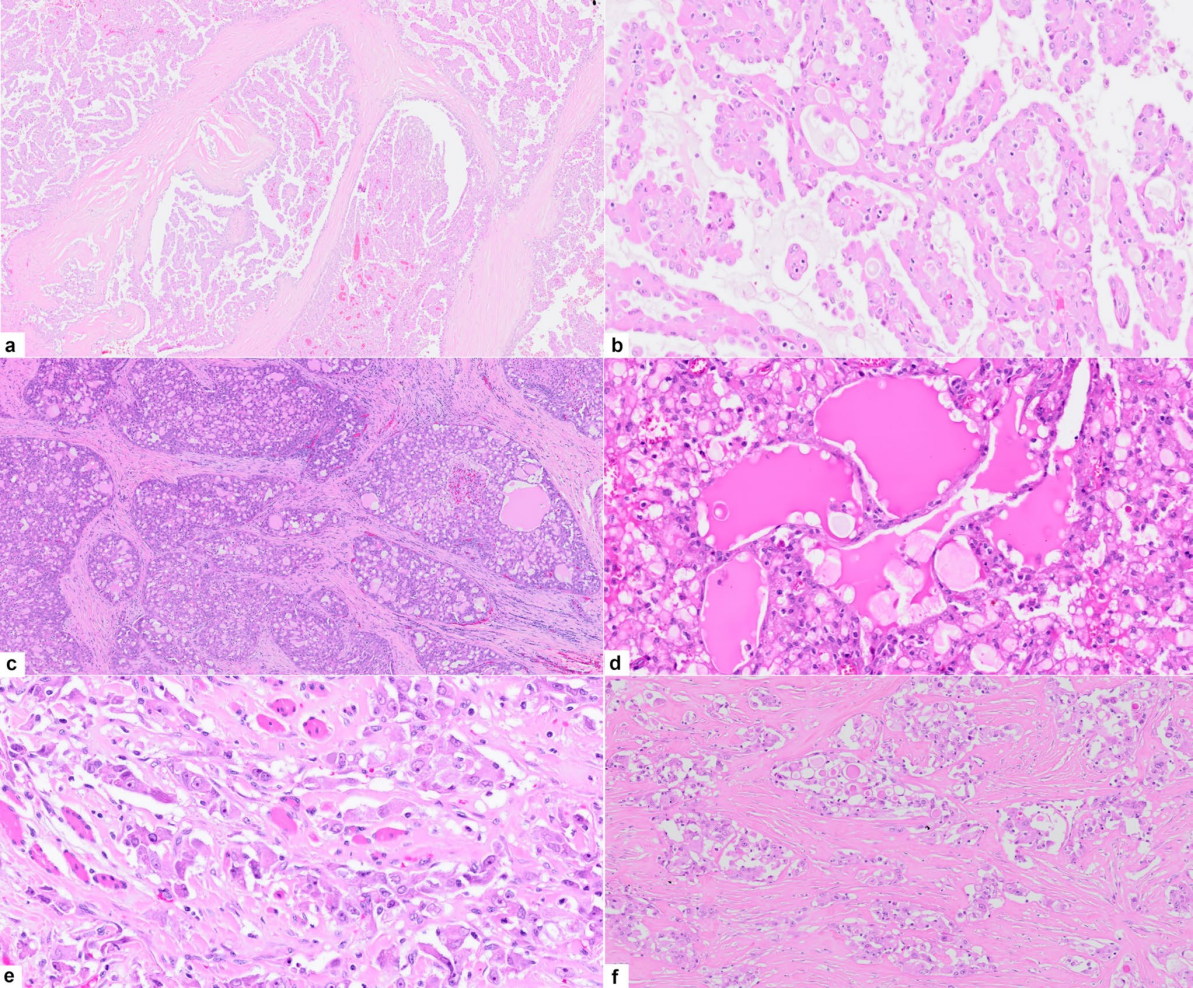
**Case 1 a-b: a**, tumor lobules composed of anastomosing papillae within cystic spaces, magnification X10 (H&E). **b**, higher magnification of the papillary pattern, magnification X40 (H&E). **Case 5: c**, a diffuse microcystic growth pattern, magnification X10 (H&E). **Case 3: d**, microcysts contain dense colloid-like secretory material, magnification X40 (H&E). **Case 7 e-f: e**, cords of pleomorphic tumor cells with prominent eosinophilic nucleoli infiltrate skeletal muscle, magnification X200 (H&E). **f**, other areas in the same tumor demonstrated a sclerosing pattern, magnification X40 (**H&E**)

**Fig. 2:**
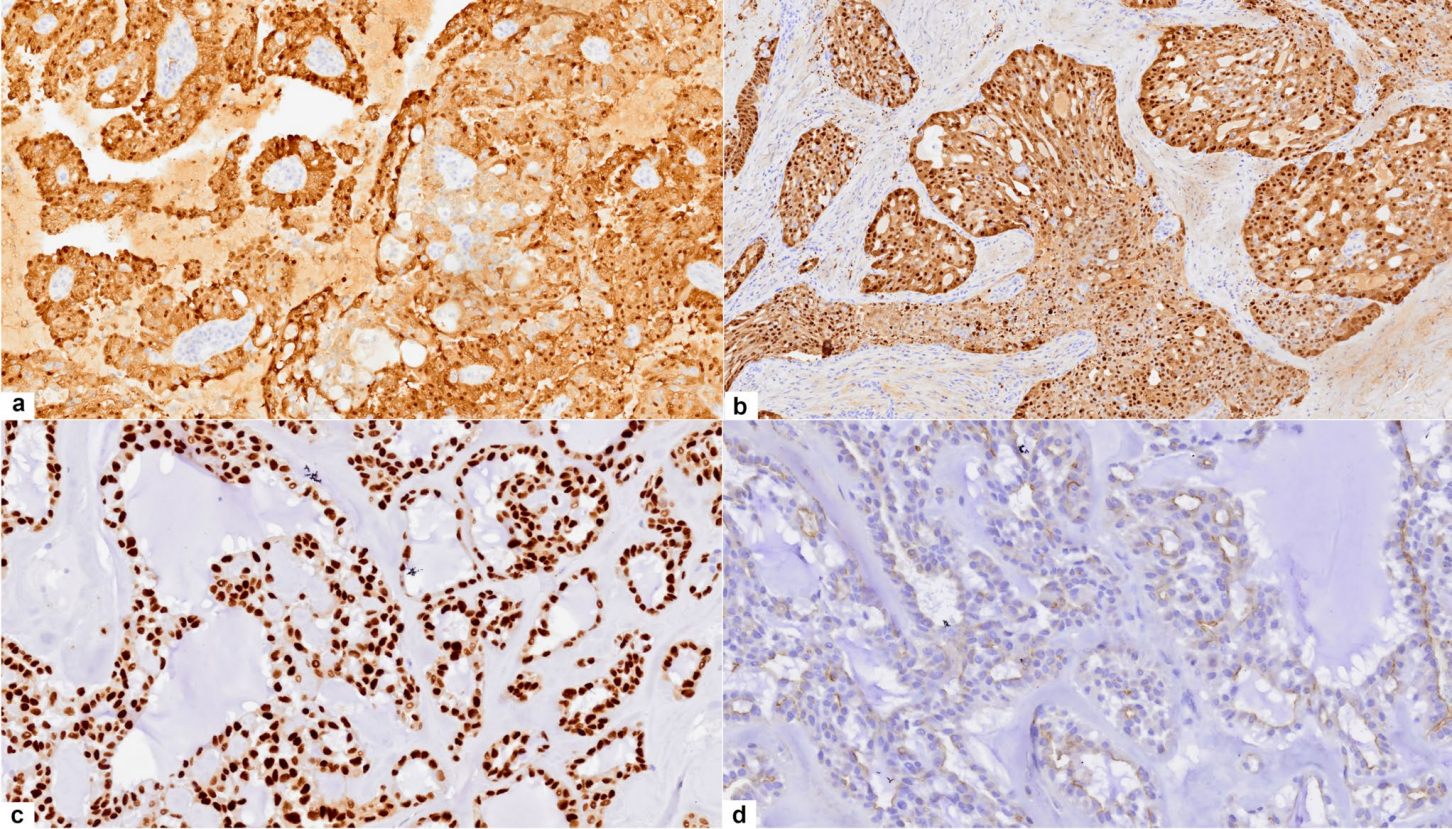
**Case 1** and **Case 5**: **a-b** immunohistochemistry for S100 showed strong diffuse nuclear and cytoplasmic staining, magnification X40. **Case 2 c-d: c**, immunohistochemistry for SOX10 showed diffuse nuclear staining, magnification X200. **d**, staining for DOG1 showed moderate immunoreactivity confined to the luminal membranes of the tumor cells, magnification X200

**Fig. 3:**
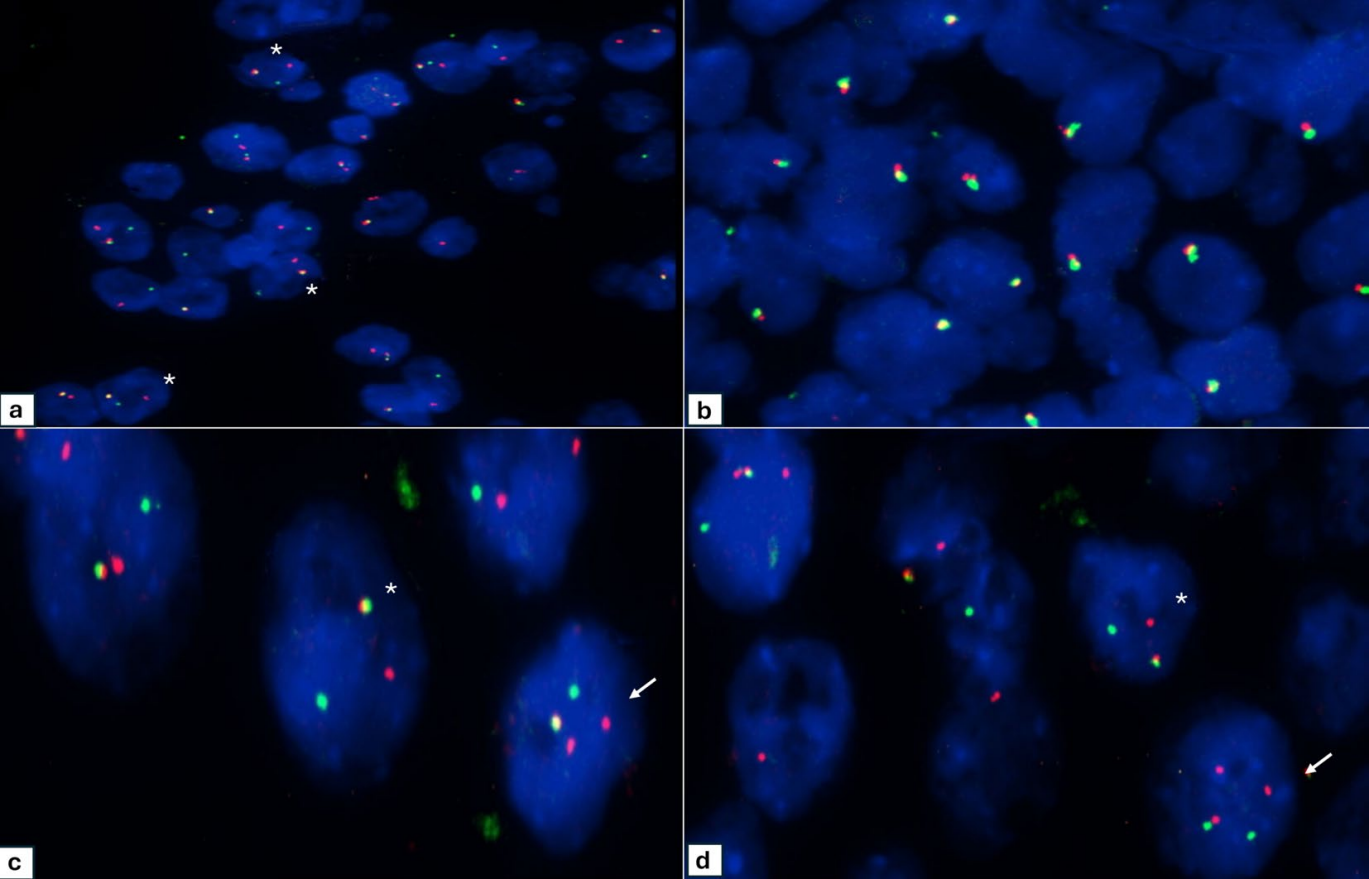
**Case 1: a** FISH analysis of *ETV6* gene rearrangement demonstrates a classic pattern of one fusion signal and one break-apart signal *(asterisk)*. **Case 2: b** all the tumor cells analyzed have an atypical pattern of loss of one copy of *ETV6* without gene rearrangement. **Case 7 c-d**: atypical *ETV6* FISH signal pattern showing one fusion signal, separated orange and green signals *(asterisk)* and gain of orange signal *(arrow)*

Histologically, all cases demonstrated a lobulated growth pattern with fibrous septa. The morphologic features of the tumor lobules comprised varied combinations of microcystic (cases 2, 5, 6 and 7), papillary (case 1 and 4), tubular (case 5), and follicular patterns (case 3) often with one pattern predominating (Fig. 1a-d). The microcysts contained homogenous eosinophilic secretions, positive for periodic acid–Schiff (PAS) with and without diastase digestion. In the papillary pattern, tumor cells typically filled prominent cystic spaces, thin fibrovascular cores were often visible within the papillary epithelial projections and some exhibited an overlapping microcystic pattern (Fig. 1b). Zymogen granules were absent, and the cytoplasm was eosinophilic to prominently vacuolated. The tumors were mitotically inert, and necrosis was absent. Except for one of the recurrent tumors (case 7), all SCs were cytologically low grade with bland round to ovoid nuclei, finely granular chromatin, and occasional small nucleoli. Local perineural invasion and isolated single lymph node metastases were identified in 2 cases (case 5 and 7), which included the cytologically high grade recurrent SC which further showed widespread skeletal muscle involvement (Fig. 1e), bone and lymphovascular invasion. Infiltrating nests of tumor cells and a fibrosclerotic stroma were also prominent in the latter case (Fig. 1f). Immunohistochemically, S100 staining was carried out on 6 cases (Table 1; Fig. 2. a, b), and all were diffusely positive. Cases 1–4 and case 7 demonstrated diffuse positivity for SOX10 (Fig. 2c), while case 2 and case 4 further showed luminal DOG1 staining only with no cytoplasmic reaction (Fig. 2d). The remaining 5 cases were all completely negative for DOG1. *ETV6* break-apart analysis was performed in all 7 cases, and the results are summarized in Table 1; Fig. 3a-d.

### Treatment and Follow-up

All 7 patients underwent surgical excision of the tumor, with one of the recurrent tumors requiring an extensive wide local excision including segmental mandibular resection, excision of the overlying skin and level II-IV neck dissection (case 7). Further treatment included subsequent total parotidectomy for close margins (< 5 mm) with elective neck dissection (case 1), and radiotherapy as an adjunct to surgery in 2 patients (case 5 and 7). Follow-up data was available in 4 cases, with a median follow up of 9.5 months post-surgery for primary (*n* = 3) and recurrent disease (*n* = 1) (Table 1).

### Literature Review

The search returned 10 manuscripts published between 2012 and 2022, describing 51 salivary gland carcinomas with an atypical *ETV6* FISH result (Table 2).

**Table 2:** Summary of literature findings on atypical *ETV6* FISH patterns in salivary gland tumors

Author	Country	*N*=	Age / Sex	Location	Diagnosis	*ETV6* FISH
Chiosea et al. 2012 [10]	USA	1	NS	Parotid	AciCC with HGT	Deletion of one fusion signal
Williams et al. 2013 [11]	USA	1	37 F	Parotid	SC	1F1O1G and extra orange signals
Patel et al. 2013 [12]	USA	1	NS	NS	SC	1F1R1G and extra red signals
Pinto et al. 2014 [13]	USA	2	83 M	Parotid	AciCC	Deletion of *ETV6* gene
			76 M	Parotid	AC (NOS)	Amplification of *ETV6* gene
Stevens et al. 2015 [14]	USA	1	NS	NS	SC	1F1O1G and extra copy of *ETV6* gene
		1	NS	Parotid	AciCC	Loss of one copy of *ETV6* gene
		11	NS	NS	HG-SDC	Extra copies of *ETV6* gene
Khurram et al. 2016 [15]	UK	2	62 M	Parotid	SC	Increased copies of *ETV6* fusion signal
			84 M	Parotid	SC	Increased copies of *ETV6* fusion signal
Naous et al. 2017 [16]	USA	1	33 M	Parotid	SC	*ETV6* 3` interstitial deletion
Ayre et al. 2019 [17]	Canada	1	NS	Parotid	SC	1F1R1G and extra red signals
Sun et al. 2022 [8]	China	27	M: F ratio 1.7:1	Parotid (*n* = 19), buccal region (*n* = 3), SMG (*n* = 1), other (*n* = 4)	SC	1F1GnR with extra red signals (*n* = 22), *ETV6* 3`deletion (*n* = 2), *ETV6* 5`deletion (*n* = 1), *ETV6* 3`deletion and *ETV6* 5` duplication (*n* = 1), 1R1G (*n* = 1)
Xu et al. 2022 [18]	USA	2	NS	NS	SC	Deletion of *ETV6* gene (*n* = 1), inversion of *ETV6* gene (*n* = 1)

FISH, fluorescence in situ hybridization; 1F1O1G, one fusion, one orange and one green signal; 1F1R1G, one fusion, one red and one green signal; F, female; M, male; NS, not specified; SMG; submandibular gland; AciCC, acinic cell carcinoma; HGT, high-grade transformation; SC, secretory carcinoma; AC (NOS) adenocarcinoma not otherwise specified; HG-SDC, high-grade salivary duct carcinoma; UK, United Kingdom; USA, United States of America

## Discussion

In this case series report, the demographic, clinical, histopathologic and *ETV6* break-apart FISH patterns of 7 salivary gland carcinomas are presented. Tumors were most frequently located in the parotid gland, followed by the buccal mucosa and submandibular gland. Four carcinomas harbored atypical *ETV6* FISH patterns, of which 3 were found in non-parotid locations, and 2 cases presented as recurrent disease. To our knowledge, this is the first case series of salivary gland carcinomas with *ETV6* molecular analysis in Africa, and wherein variations in *ETV6* break-apart FISH patterns are compared to previous reports.

The association of atypical *ETV6* FISH signals with epidemiological, clinical and prognostic parameters in salivary gland carcinomas has not been fully characterized. In a review of the literature, 36 cases of salivary SC showing an atypical *ETV6* FISH pattern were identified (Table 2). Nine cases were from Western countries [11–18] and 27 cases were from China [8]. There was a male predilection, and the parotid gland was the most common site of involvement. The most frequent atypical FISH pattern was *ETV6* rearrangement with gain of red/orange signals (69.4%, 25/36; Table 2). Three cases exhibited changes in *ETV6* copy number either in isolation [15, 18] or in combination with classic *ETV6* break-apart FISH patterns [14]. Less frequently encountered *ETV6* molecular aberrations in salivary SC included an *ETV6* 3` interstitial deletion [8, 16] and inversion of the *ETV6* gene [18]. Among salivary gland tumors, although *ETV6* rearrangements have been exclusively associated with SC [5], alterations in *ETV6* copy number have been reported in other malignant salivary gland neoplasms (Table 2). In a comparative study assessing *ETV6* gene status in salivary gland tumors, almost all cases of high-grade salivary duct carcinomas (11/12, 91.7%) demonstrated increased *ETV6* fusion signals, indicative of gain of the *ETV6* gene [14]. By contrast, deletion of one copy of the *ETV6* gene has been observed in isolated cases of acinic cell carcinoma [10, 13, 14]. Since most of the salivary gland carcinomas with atypical *ETV6* FISH patterns were identified when screening for SC among its mimics in surgical pathology archives, there is limited individual patient information currently available for evaluation of the clinical implications of alterations in *ETV6* copy number in these tumors.

By contrast, there has been a greater focus on the prognostic value of the more prevalent atypical pattern of *ETV6* break-apart with gain of red/orange signals in SC. Although the exact significance of this phenomenon in salivary SC is not known, the literature findings provide some insight into the clinicopathologic trends associated with this aberrant *ETV6* FISH pattern. Sun et al. [8] found a higher proportion of salivary SC cases with increase of the red signal associated with necrosis, neural invasion and a higher proliferation index compared to SCs with the classic *ETV6* FISH pattern. In an analysis of the clinical behavior of SC of the major salivary glands, Ayre et al. [17] reported a recurrent SC that was found to have facial nerve invasion, extraglandular extension, and a positive lymph node. Although mitotic figures were infrequent, and there was no evidence of necrosis or high-grade transformation, this case demonstrated extra red signals on *ETV6* FISH analysis. The patient experienced multiple subsequent recurrences in the parotid bed and the surrounding skin of the ear and neck, and ultimately died of lung metastases and uncontrolled regional disease. A pattern of lymph node spread, perineural infiltration, vascular, skin, skeletal muscle and bone invasion was also seen in case 7 which harbored *ETV6* rearrangement with gain of orange signals as described above. There are reports of similar observations in SCs presenting outside the salivary glands. An unbalanced *ETV6* translocation was recently reported in 2 cases of SC presenting in the sinonasal region and where high-grade transformation was documented in one of the cases and focal geographic necrosis in the other [19]. Furthermore, in the first reported case of thyroid SC with diffuse metastatic disease at presentation, it is noteworthy that while 22% of the tumor cells had a typical *ETV6* rearrangement, the remaining tumor cells showed one intact gene and one rearranged gene with gain of the red signal [20]. This case also showed prominent necrosis, multiple foci of extrathyroidal extension, an aggressive clinical course and death of the patient from extreme airway compromise [20].

In a series of secretory breast carcinoma with the *ETV6::NTRK3* fusion (confirmed on RT-PCR), the morphologic features of the SCs carrying a balanced *ETV6* reciprocal translocation (8/10) were typically low grade. However, 2 cases showed unbalanced *ETV6* break-apart with gain of the oncogenic derivative (red signal) and with corresponding high histologic grade (moderate to marked nuclear atypia, vascular invasion and tumor necrosis) [21]. Lastly, in a single report of a primary pulmonary SC, FISH testing for *ETV6* rearrangement demonstrated a clonal variant with an extra 5′ *ETV6* signal pattern (83% of tumor cells). This case also demonstrated aggressive features; notably an increased mitotic count (6/10 high-power fields) but without high grade cytology, and advanced-stage disease as evidenced by lymph node metastases, visceral and pleural invasion [22]. Although the numbers of published cases thus far are low, it appears that unbalanced *ETV6* break-apart with an extra 5’*ETV6* signal pattern may be associated with more infiltrative histologic features and less favourable clinical outcomes in SC.

Two of the cases in the current series exhibited loss of one *ETV6* fusion signal without rearrangement (Table 1). One of the cases represented a recurrent tumor in the buccal mucosa that was originally diagnosed as an acinic cell carcinoma. By immunohistochemistry, the tumor cells showed diffuse S100 and SOX10 positivity, while luminal DOG1 staining without a cytoplasmic reaction was also observed in this case. Interestingly, Stevens et al. [14] reported a similar pattern of apical membranous DOG1 staining in 10/12 SCs and wherein typical *ETV6* rearrangements were documented in 10/11 cases tested. This DOG1 staining pattern contrasted with the diffuse, strong, cytoplasmic and canalicular DOG1 expression that was demonstrated in acinic cell carcinomas in the same study. In another study, DOG1 expression was noted along the luminal surface of tumor cells in one-third of salivary SCs with atypical FISH signal patterns using break-apart and *ETV6::NTRK3* fusion probes [8]. Given the expanding molecular profile of SC these cases appear to represent a subset of salivary SC with divergent molecular findings.

In this cohort, whilst the overall histomorphologic and immunohistochemical findings of those cases without typical 1F1O1G *ETV6* rearrangements were considered consistent with SC, orthogonal testing for an *ETV6::NTRK3* fusion gene or other associated gene fusion was not available to confirm the diagnosis. The possibility of an intercalated duct type intraductal carcinoma was, however, considered unlikely as a rim of myoepithelial cells surrounding the tumor lobules was not visualized by p63 immunostaining in these cases. Nevertheless, given the limitations of this study which includes the lack of testing for NR4A3 (NOR1) or NR4A2 (NURR1) expression, a non-secretory salivary carcinoma cannot be entirely excluded in those cases without typical 1F1O1G rearrangements. The nosology of salivary gland tumors harboring deletion of one *ETV6* fusion signal and demonstrating apical membranous staining for DOG1 around tumor lumina thus remains to be clarified. Future investigations are also needed to determine whether the nature and frequency of *ETV6* genetic abnormalities in salivary SC varies by geographic region or are instead acquired in association with disease progression.

## Conclusion

Among the 40 cases of salivary SC showing variations in *ETV6* break-apart FISH patterns as reported in the literature and including 4 cases from the current series, *ETV6* rearrangement with duplication of the *ETV6* 5`end (1F1GnR, *n* ≥ 2) represented the most common (26/40; 65%) variation. Larger studies are required to confirm the link of duplication of the distal/telomeric *ETV6* probe with a more aggressive disease course in salivary SC.

## Data Availability

No datasets were generated or analysed during the current study.
